# Loss of endogenous thymosin β_4_ accelerates glomerular disease

**DOI:** 10.1016/j.kint.2016.06.032

**Published:** 2016-11

**Authors:** Elisavet Vasilopoulou, Maria Kolatsi-Joannou, Maja T. Lindenmeyer, Kathryn E. White, Michael G. Robson, Clemens D. Cohen, Neil J. Sebire, Paul R. Riley, Paul J. Winyard, David A. Long

**Affiliations:** 1 Developmental Biology and Cancer Programme, UCL Institute of Child Health, London, United Kingdom; 2 Nephrological Center, Medical Clinic and Policlinic IV, University of Munich, Munich, Germany; 3 EM Research Services, University of Newcastle, Newcastle upon Tyne, United Kingdom; 4 MRC Centre for Transplantation, King’s College London, London, United Kingdom; 5 Department of Physiology, Anatomy and Genetics, University of Oxford, Oxford, United Kingdom

**Keywords:** cytoskeleton, fibrosis, glomerulus, inflammation, podocyte

## Abstract

Glomerular disease is characterized by morphologic changes in podocyte cells accompanied by inflammation and fibrosis. Thymosin β_4_ regulates cell morphology, inflammation, and fibrosis in several organs and administration of exogenous thymosin β_4_ improves animal models of unilateral ureteral obstruction and diabetic nephropathy. However, the role of endogenous thymosin β_4_ in the kidney is unknown. We demonstrate that thymosin β_4_ is expressed prominently in podocytes of developing and adult mouse glomeruli. Global loss of thymosin β_4_ did not affect healthy glomeruli, but accelerated the severity of immune-mediated nephrotoxic nephritis with worse renal function, periglomerular inflammation, and fibrosis. Lack of thymosin β_4_ in nephrotoxic nephritis led to the redistribution of podocytes from the glomerular tuft toward the Bowman capsule suggesting a role for thymosin β_4_ in the migration of these cells. Thymosin β_4_ knockdown in cultured podocytes also increased migration in a wound-healing assay, accompanied by F-actin rearrangement and increased RhoA activity. We propose that endogenous thymosin β_4_ is a modifier of glomerular injury, likely having a protective role acting as a brake to slow disease progression.

End-stage renal failure is a devastating condition requiring lifelong dialysis and transplantation and a risk factor for all-cause mortality and cardiovascular disease.^1^ Many cases are due to disruption of the glomerular filtration barrier, consisting of epithelial podocytes, the endothelium, the mesangium, and the glomerular basement membrane.^2^ Podocytes have a unique shape maintained by a complex cytoskeleton,^3^ with branched foot process extensions that abut each other at slit diaphragms. During glomerular injury, the podocyte architecture is perturbed, resulting in defective filtration and proteinuria,^2^, ^3^, ^4^ often with inflammatory components characterized by leukocyte infiltration followed by glomerulosclerosis and tubulointerstitial fibrosis.^5^

Thymosin β_4_ (Tmsb4x) is a naturally occurring peptide. It is the major G-actin sequestering protein in mammalian cells,^6^ with critical roles in maintaining the cell cytoskeleton. In animal models, exogenous Tmsb4x has beneficial effects in diverse pathologies including myocardial infarction,^7^ stroke,^8^ dry eye,^9^ and inflammatory lung disease,^10^ and there are clinical trials assessing Tmsb4x treatment in wound healing and cardioprotection.^11^ The utility of Tmsb4x in these pathologies has been attributed to modulation of several cellular functions including cell motility,^12^ differentiation,^13^ survival,^14^ angiogenesis,^15^ inflammation,^16^ and fibrosis.^10^

Recent studies investigated exogenous Tmsb4x as a treatment for kidney disease.^17^ Tmsb4x reduced renal tubulointerstitial fibrosis after unilateral ureteral obstruction in mice, potentially through decreasing plasminogen activator inhibitor-1 expression and dampening transforming growth factor-β_1_ signaling.^18^ In *KK Cg-Ay/J* mice, a model of type 2 diabetes mellitus, daily Tmsb4x treatment for 3 months reduced albuminuria and attenuated renal pathology.^19^ Furthermore, *N*-acetyl-seryl-aspartyl-lysyl-proline, the N-terminal tetrapeptide generated by Tmsb4x cleavage,^20^ has beneficial effects on fibrosis and inflammation in unilateral ureteral obstruction, remnant kidneys, diabetic nephropathy, and glomerulonephritis.^18^, ^21^, ^22^, ^23^

*Tmsb4x* transcripts are detectable by *in situ* hybridization in developing and adult glomeruli,^24^ with strong expression in podocytes.^24^, ^25^ Furthermore, in rat remnant kidneys, proteomic analysis of laser-capture dissected glomeruli demonstrated significantly increased Tmsb4x in sclerotic versus normal glomeruli.^26^ Despite such evidence of expression and beneficial renal effects, the functional importance of endogenous Tmsb4x in the kidney during health and disease is completely unknown.

In this study, we confirmed that Tmsb4x is highly expressed in the kidney glomerulus, predominantly in podocytes. Using global *Tmsb4x* knockout mice,^27^ we demonstrated that endogenous Tmsb4x was dispensable in healthy glomeruli. Furthermore, in an experimental model of glomerular damage, lack of *Tmsb4x* worsened disease progression by (i) enhancing podocyte migration, facilitating their redistribution from the glomerular tuft to the Bowman capsule, and (ii) increasing periglomerular inflammation and interstitial fibrosis. Thus, we provide the first evidence that endogenous Tmsb4x is critical in the progression of glomerular disease.

## Results

### Tmsb4x is expressed in mouse glomerular podocytes

**Figure S1:**
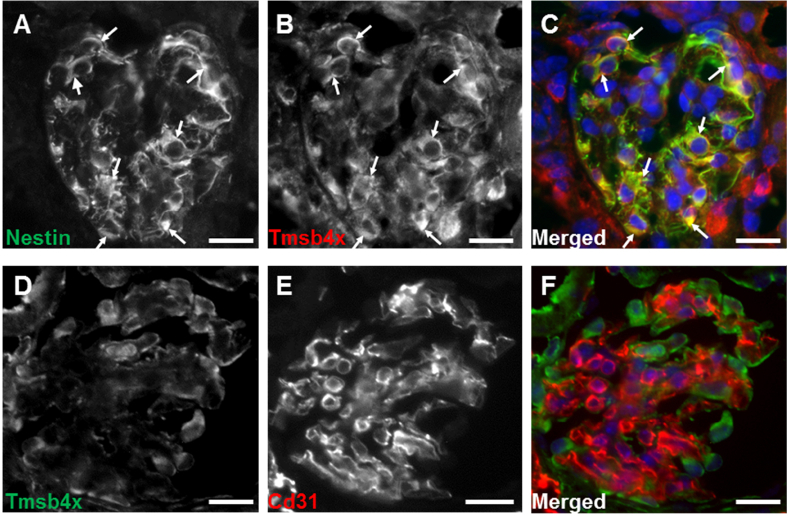
Representative images of nestin (**A**) and Tmsb4x (**B**) staining in the mouse adult wild-type glomerulus visualized by fluorescent microscopy. (**C**) Merged images showing Tmsb4x (red) and nestin (green) staining; areas of colocalization are indicated by arrows. Representative images of Tmsb4x (**D**) and Cd31 (**E**) staining in the mouse adult wild-type glomerulus visualized by fluorescent microscopy. (**F**) Merged images showing Tmsb4x (green) and Cd31 (red) staining. Bar = 20 μm.

**Figure 1:**
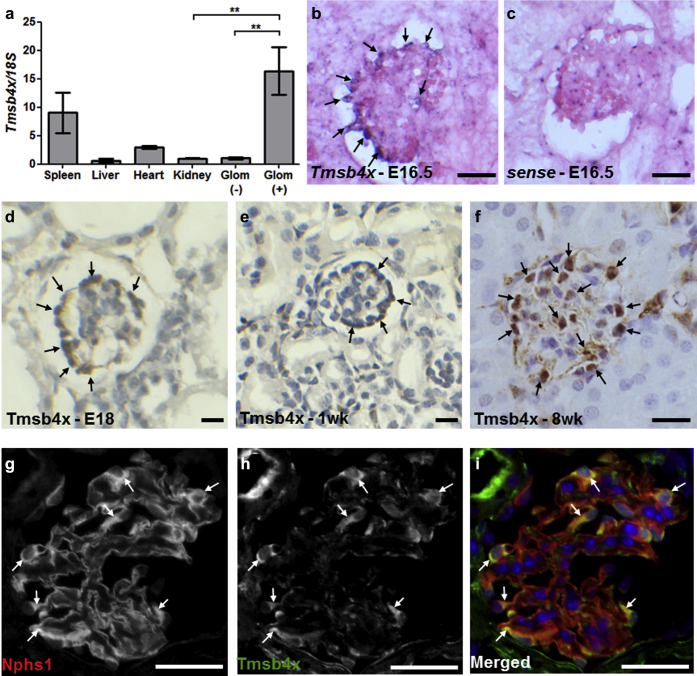
***Tmsb4x* expression in the mouse glomerulus.** (**a**) Quantification of thymosin β_4_ (*Tmsb4x*) mRNA levels in adult mouse spleen, liver, heart, and kidney by quantitative reverse transcriptase polymerase chain reaction. *Tmsb4x* expression was also quantified in glomeruli-depleted [glom (-)] and glomeruli-enriched [glom (+)] kidney homogenates. The bars represent the mean of 3 samples ± SEM. (**b,c**) *In situ* hybridization for *Tmsb4x* on embryonic day (E) 16.5 mouse kidney sections. Cells positive for *Tmsb4x* are indicated by arrows. Immunohistochemistry for Tmsb4x in the mouse glomerulus at E18 (**d**), 1 week (**e**), and 8 weeks (**f**) of age. Cells positive for Tmsb4x are indicated by arrows. Representative images of Tmsb4x (**g**) and nephrin (Nphs1) (**h**) staining in the mouse adult wild-type glomerulus visualized by fluorescent microscopy. (**i**) Merged images showing Nphs1 (red) and Tmsb4x (green) staining; areas of colocalization are indicated by arrows. Bar = 20 μm. **P ≤ 0.01.

*Tmsb4x* mRNA levels were assessed in the spleen, liver, heart, and whole kidney of healthy adult mice. The highest transcript levels were found in the spleen with ∼10 times less Tmsb4x in the kidney (Figure 1a). Using Dynabead perfusion (Thermo Fisher Scientific, Waltham, MA),^28^ we isolated glomeruli and found that *Tmsb4x* levels were significantly enriched in glomeruli compared with the rest of the kidney (Figure 1a). *In situ* hybridization detected *Tmsb4x* expression in immature glomeruli of embryonic day 16.5 developing kidneys, predominantly in podocytes (Figure 1b and c). The protein expression of Tmsb4x was also assessed by immunohistochemistry, and we found strong localization in glomerular podocytes at embryonic day 18 (Figure 1d). This expression pattern was maintained in 1-week-old postnatal (Figure 1e) and 8-week-old adult kidneys (Figure 1f). Tmsb4x podocyte expression was further confirmed by colocalization of Tmsb4x with nephrin (Nphs1), a slit diaphragm component^29^ (Figure 1g–i), and nestin, an intermediate filament protein expressed in mature podocytes^30^ (Supplementary Figure S1A–C). In contrast, Tmsb4x did not colocalize with the pan-endothelial marker Cd31 (Supplementary Figure S1D–F).

### Lack of endogenous *Tmsb4x* has no effect on healthy glomeruli

**Figure S2:**
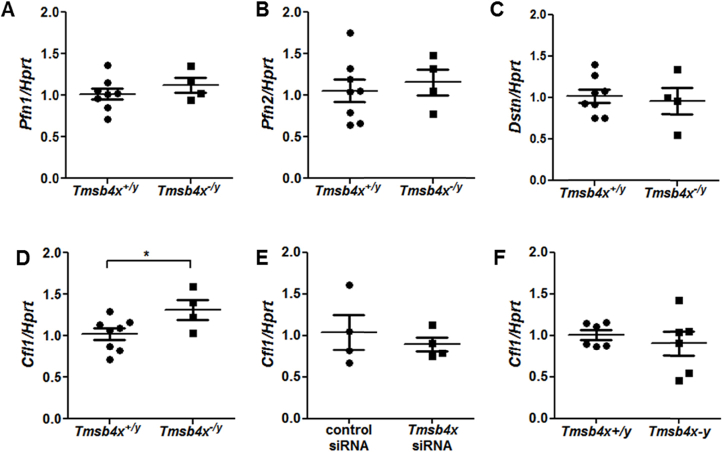
Quantification of profilin 1(*Pfn1*) (**A**), profilin 2 (*Pfn2*) (**B**), destrin (*Dstn*) (**C**), and cofilin 1 (*Cfl1*) (**D**) mRNA in whole-kidney homogenates of *Tmsb4x*^*+/y*^ (*n* = 8) and *Tmsb4x*^*−/y*^ (*n* = 4) mice by quantitative reverse transcriptase polymerase chain reaction. Quantification of cofilin 1 (*Cfl1*) mRNA in podocytes 48 hours after transfection with control siRNA or siRNA targeting *Tmsb4x* (*n* = 4) (**E**) and in podocytes isolated from *Tmsb4x*^*+/y*^ or *Tmsb4x*^*−/y*^ mice (*n* = 6) (**F**). Data are presented as mean ± SEM. *P < 0.05.

**Figure S3:**
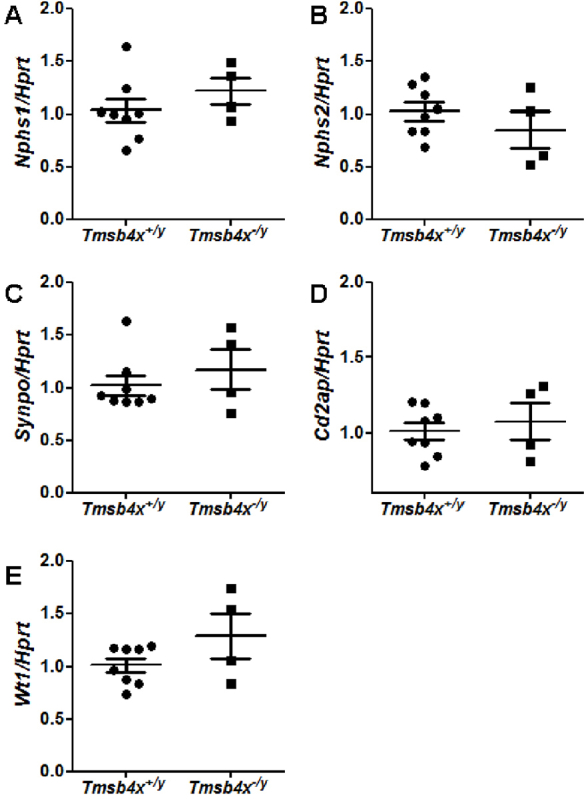
Quantification of nephrin (*Nphs1;***A**), podocin (*Nphs2*; **B**), synaptopodin (*Synpo*; **C**), cd2 associated protein (*Cd2ap*) (**D**) and Wilms tumor 1 (*Wt1*) (**E**) mRNA in whole-kidney homogenates of *Tmsb4x*^*+/y*^ (*n* = 8) and *Tmsb4x*^*−/y*^ (*n* = 4) mice by quantitative reverse transcriptase polymerase chain reaction. Data are presented as mean ± SEM.

**Figure 2:**
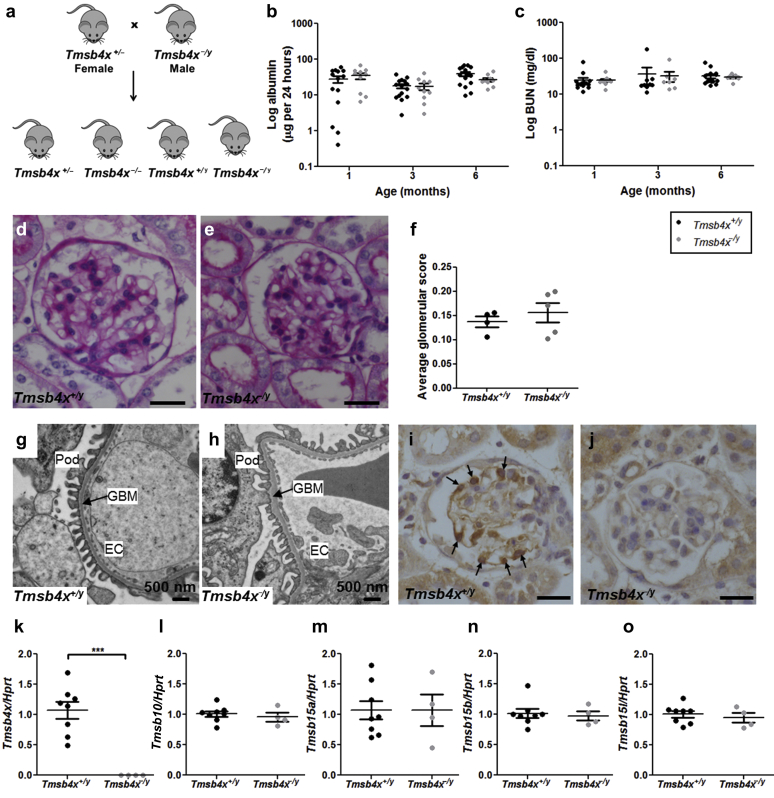
**Renal function and glomerular morphology in *Tmsb4x***^***+/y***^**and *Tmsb4x***^***−/y***^**mice.** (**a**) Breeding scheme: heterozygous female mice (*Tmsb4x*^*+/−*^) were bred with hemizygous null male mice (*Tmsb4x*^*−/y*^). Male wild-type (*Tmsb4x*^*+/y*^) and *Tmsb4x*^*−/y*^ mice were compared in all subsequent experiments. (**b**) Twenty-four hour albumin excretion in urine of *Tmsb4x*^*+/y*^ (*n* = 14–15) and *Tmsb4x*^*−/y*^ (*n* = 9–10) mice collected at 1, 3, and 6 months of age. Data were log-transformed before analysis. (**c**) Blood urea nitrogen (BUN) concentration in *Tmsb4x*^*+/y*^ (n = 9–15) and *Tmsb4x*^*−/y*^ mice (*n* = 7–9) at 1, 3, and 6 months of age. Data were log-transformed before analysis. Representative images of periodic acid–Schiff staining of paraffin-embedded sections from *Tmsb4x*^*+/y*^ (**d**) and *Tmsb4x*^*−/y*^**(e**) kidneys. Bar = 20 μm. The glomerular score (**f**) was quantified as explained in the Methods section (*Tmsb4x*^*+/y*^: *n* = 4; *Tmsb4x*^*−/y*^: *n* = 5). Representative images of the glomerular architecture of *Tmsb4x*^*+/y*^ (**g**) and *Tmsb4x*^*−/y*^ (**h**) kidneys assessed by transmission electron microscopy. An average of 5 glomeruli were examined per animal (Tmsb4x^+/y^, *n* = 4; Tmsb4x^−*/*y^, *n* = 3). Representative images of immunohistochemistry for thymosin β_4_ (Tmsb4x) on paraffin-embedded sections from *Tmsb4x*^*+/y*^ (**i**) and *Tmsb4x*^*−/y*^ (**j**) kidneys from 6-month-old mice. Tmsb4x-positive cells are indicated by arrows. Note: nonspecific staining in tubules in *Tmsb4x*^*+/y*^ mice and *Tmsb4x*^*−/y*^ mice. Quantification of *Tmsb4x* (**k**), *Tmsb10* (**l**), *Tmsb15a* (**m**), *Tmsb15b* (**n**), and *Tmsb15l* (**o**) mRNA levels in whole-kidney homogenates of *Tmsb4x*^*+/y*^ (*n* = 8) and *Tmsb4x*^*−/y*^ (*n* = 4) mice by quantitative reverse transcriptase polymerase chain reaction. Data are presented as mean ± SEM. ***P ≤ 0.001. Bar = 20 μm. EC, endothelial cell; GBM, glomerular basement membrane; HPRT, hypoxanthine-guanine phosphoribosyltransferase; Pod, podocyte; Tmsb4x, thymosin β_4_; Tmsb10, thymosin β10; Tmsb15a, thymosin β15a; Tmsb15b, thymosin β15b; Tmsb15b1, thymosin β15b-like.

We examined mice with a global loss of *Tmsb4x*^27^ to assess the importance of endogenous *Tmsb4x* in healthy glomeruli. As Tmsb4x is mapped to the X chromosome,^31^ we crossed hemizygous null male mice (*Tmsb4x*^*−/y*^) with heterozygous *Tmsb4x*^*+/−*^ adult female mice (Figure 2a). We found no lethal developmental abnormalities with the offspring conforming to Mendelian ratios (Supplementary Table S1). *Tmsb4x*^*−/y*^ mice had albumin excretion (Figure 2b) and blood urea nitrogen (Figure 2c) levels similar to those of male wild-type *Tmsb4x*^*+/y*^ mice at the ages of 1, 3, and 6 months. There was no difference in body weight at 1 (*Tmsb4x*^*+/y*^ = 23.8 ± 0.7 g; *Tmsb4x*^*−/y*^ = 23.4 ± 0.4 g), 3 (*Tmsb4x*^*+/y*^ = 30.0 ± 0.5 g; *Tmsb4x*^*−/y*^ = 29.2 ± 0.7 g), or 6 (*Tmsb4x*^*+/y*^ = 35.8 ± 1.0 g; *Tmsb4x*^*−/y*^ = 35.3 ± 1.6 g) months of age. Using semiquantitative analysis of light microscopy images, we found no differences in glomerular morphology between 6-month-old *Tmsb4x*^*+/y*^ and *Tmsb4x*^*−/y*^ mice (Figure 2d–f). This was confirmed by transmission electron microscopy with normal foot process architecture, laminar structure of the basement membrane, and the presence of endothelial fenestrae in both *Tmsb4x*^*+/y*^ and *Tmsb4x*^*−/y*^ mice (Figure 2g and h). We demonstrated the loss of Tmsb4x protein in glomeruli of *Tmsb4x*^*−/y*^ mice compared with *Tmsb4x*^*+/y*^ (Figure 2i and j), along with undetectable *Tmsb4x* mRNA levels in whole kidneys (Figure 2k). We examined whether there was any compensation for the lack of *Tmsb4x* by other β-thymosins, but found no changes in the renal mRNA levels of *Tmsb10*, *Tmsb15a*, *Tmsb15b*, and *Tmsb15l* (Figure 2l–o). We also assessed mRNA levels of genes involved in actin polymerization and found no differences in profilin (*Pfn*) 1 and 2 and destrin (*Dstn*) between *Tmsb4x*^*−/y*^ and *Tmsb4x*^*+/y*^ mice (Supplementary Figure S2A–C). In contrast, cofilin 1 (*Cfl1*) mRNA levels were significantly increased in *Tmsb4x*^*−/y*^ kidneys by ∼30% compared with *Tmsb4x*^*+/y*^ mice (Supplementary Figure S2D). We specifically examined *Cfl1* mRNA levels in podocytes and found no change after knock down of endogenous *Tmsb4x* by small, interfering RNA (siRNA) (Supplementary Figure S2E). Furthermore, there was no difference in *Cfl1* mRNA levels in primary podocytes isolated from *Tmsb4x*^*−/y*^ and *Tmsb4x*^*+/y*^ mice (Supplementary Figure S2F). Finally, the mRNA levels of genes important for podocyte function (*Nphs1*, *Nphs2*, *Synpo*, *Cd2ap*, and *Wt1*) were unchanged in *Tmsb4x*^*−/y*^ and *Tmsb4x*^*+/y*^ kidneys (Supplementary Figure S3A–E).

### Lack of endogenous *Tmsb4x* worsens renal function and glomerular injury in nephrotoxic serum nephritis

**Figure S4:**
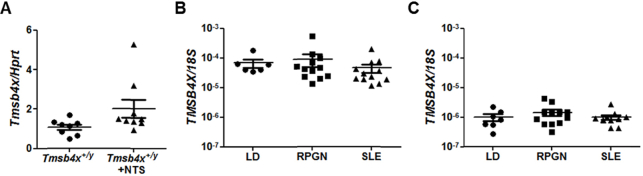
(**A**) Quantification of *Tmsb4x* mRNA levels in whole-kidney homogenates of *Tmsb4x*^*+/y*^ (*n* = 8) and *Tmsb4x*^*+/y*^ + NTS (*n* = 9) mice by quantitative reverse transcriptase polymerase chain reaction. (**B** and **C**) Quantification of *TMSB4X* mRNA levels in homogenates from human glomeruli (**B**) or tubulointerstitium (**C**). LD, living donor; RPGN, rapidly progressive glomerulonephritis; SLE, lupus nephritis. Data are presented as mean ± SEM.

**Figure 3:**
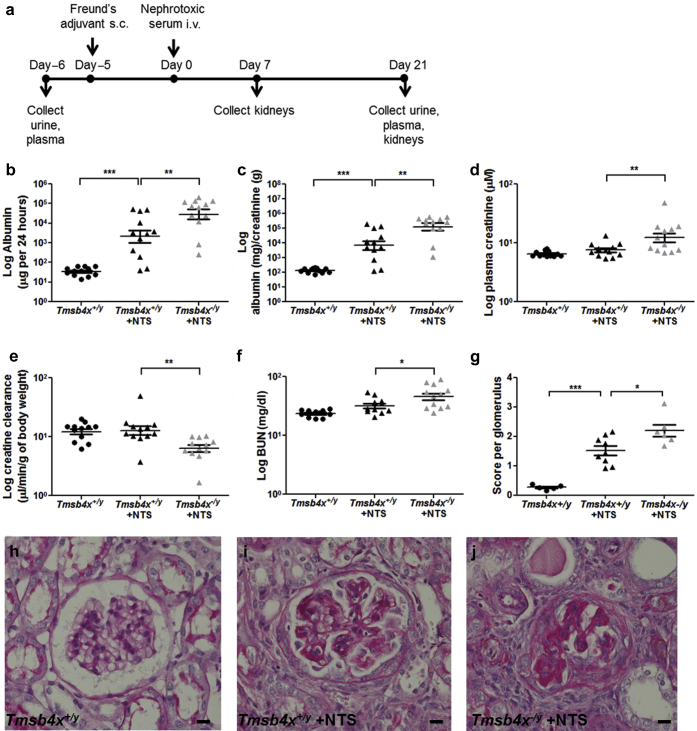
**Renal function in *Tmsb4x***^***+/y***^**and *Tmsb4x***^***−/y***^**mice after the induction of nephrotoxic nephritis.** (**a**) Outline of experimental strategy. Twenty-four–hour albumin excretion in urine (**b**), urinary albumin to urinary creatinine ratio **(c)**, plasma creatinine concentration (**d**), creatinine clearance (**e**), and blood urea nitrogen (BUN) concentration (**f**) of *Tmsb4x*^*+/y*^ and *Tmsb4x*^*−/y*^ mice. Samples were collected before immunization with Freund’s adjuvant (*Tmsb4x*^*+/y*^, control group) and 21 days after administration of nephrotoxic serum (NTS) (*Tmsb4x*^*+/y*^ + NTS and *Tmsb4x*^*−/y*^ + NTS); *n* = 12 in each group. Data were log-transformed before analysis and presented as mean ± SEM. (**g**) Glomerular score was quantified as described in the Methods section in *Tmsb4x*^*+/y*^ (*n* = 5), *Tmsb4x*^*+/y*^ + NTS (*n* = 9), and *Tmsb4x*^*−/y*^ + NTS (*n* = 6) mice. Data are presented as mean ± SEM. Representative images of periodic acid–Schiff staining in glomeruli from control mice (*Tmsb4x*^*+/y*^; **h**) and mice administered NTS; *Tmsb4x*^*+/y*^ + NTS (**i**) and *Tmsb4x*^*−/y*^ + NTS (**j**) are shown. Bar = 20 μm. *P ≤ 0.05, **P ≤ 0.01, ***P ≤ 0.001. s.c., subcutaneous; Tmsb4x, thymosin β_4_.

Our results suggest that the lack of *Tmsb4x* does not affect the function and morphology of healthy glomeruli. Therefore, we investigated whether *Tmsb4x* has a role in glomerular disease. We utilized the nephrotoxic serum (NTS) nephritis model, which replicates some of the pathologic features of human crescentic glomerulonephritis.^32^ NTS nephritis involves the injury of intrinsic glomerular cells, including podocytes, as well as leukocyte infiltration, glomerulosclerosis, and tubulointerstitial fibrosis,^33^ processes in which Tmsb4x has been implicated.^10^, ^16^ We predicted that the lack of global *Tmsb4x* may exacerbate NTS nephritis severity and examined this in 3-month-old *Tmsb4x*^*−/y*^ and *Tmsb4x*^*+/y*^ mice (Figure 3a). Albuminuria and the albumin/creatinine ratio were significantly increased in *Tmsb4x*^*+/y*^ mice 21 days after NTS administration compared with the levels before immunization (*P* < 0.001 in both cases) (Figure 3b and c). Strikingly, both albuminuria and the albumin/creatinine ratio were further enhanced by ∼5- and 7-fold, respectively, when NTS was injected in *Tmsb4x*^*−/y*^ mice compared with *Tmsb4x*^*+/y*^ mice (*P* < 0.01 in both cases) (Figure 3b and c). Administration of NTS to *Tmsb4x*^*−/y*^ mice also significantly increased plasma creatinine (*P* < 0.01) (Figure 3d), impaired creatinine clearance (*P* < 0.01) (Figure 3e), and raised the blood urea nitrogen level (*P* < 0.05) (Figure 3f) compared with *Tmsb4x*^*+/y*^ mice with NTS nephritis. We found no difference in *Tmsb4x* levels in whole kidneys obtained from *Tmsb4x*^*+/y*^ mice without disease or administered NTS (Supplementary Figure S4A). Furthermore, in kidney biopsy specimens obtained from patients with either rapidly progressive glomerulonephritis or lupus nephritis, there was no change in glomerular or tubulointerstitial *TMSB4X* mRNA levels compared with living donor control kidneys (Supplementary Figure S4B and C).

**Figure S5:**
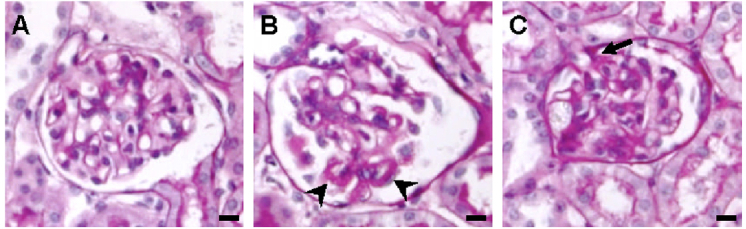
Representative images of the histology observed 7 days after NTS administration showing a normal glomerulus (**A**), a glomerulus with hyaline deposits indicated by arrowheads (**B**), and a glomerulus with a bridge formed between the glomerular tuft and the Bowman capsule indicated by an arrow (**C**). Bar = 20 μm.

**Figure S6:**
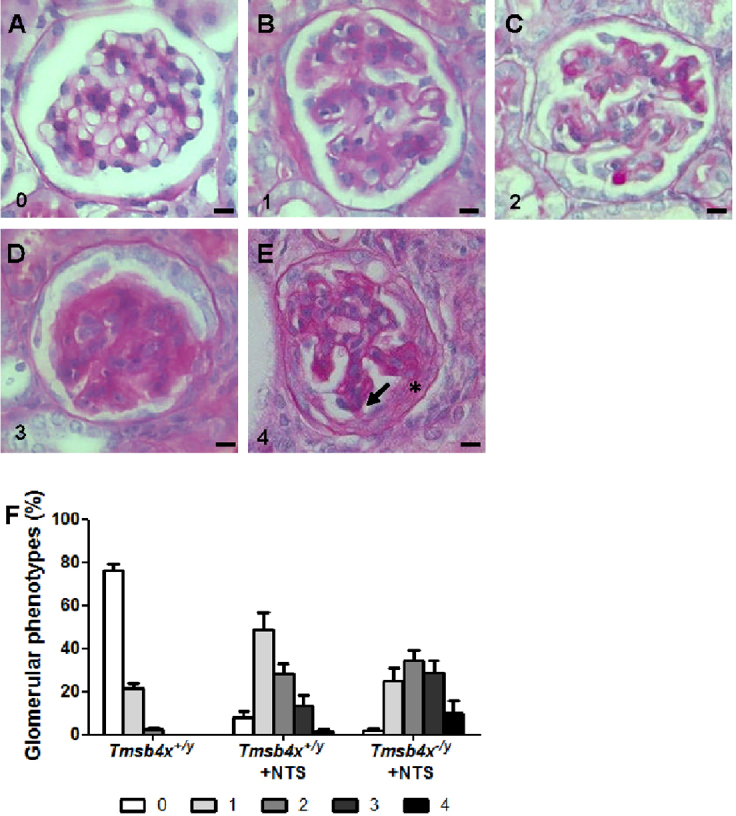
Representative images of the different scoring categories for glomerular injury showing a normal glomerulus, score 0 (**A**); a glomerulus with increased mesangial matrix deposition and some loss of capillary loops, score 1 (**B**); a glomerulus with increased mesangial matrix deposition and focal areas of sclerosis, score 2 (**C**); a glomerulus with >50% sclerotic area and very few capillary loops, score 3 (**D**); and a sclerotic glomerulus with an epithelial hyperplastic lesion (*) and a bridge formed between the glomerular tuft and the Bowman capsule indicated by an arrow, score 4 (**E**). Bar = 20 μm. (**F**) Proportions of glomeruli in the different scoring categories of glomerular injury in *Tmsb4x*^*+/y*^ (*n* = 5), *Tmsb4x*^*+/y*^ + NTS (*n* = 9), and *Tmsb4x*^*−/y*^ + NTS (*n* = 6) mice. Bars represent the mean ± SEM.

Seven days after NTS administration, we observed mild glomerular injury in Tmsb4x^*−/y*^ and *Tmsb4x*^*+/y*^ mice, with some glomeruli containing hyaline deposits, increased mesangial matrix, and occasional adhesion of the glomerular tuft to the Bowman capsule (Supplementary Figure S5A–C). After 21 days, there was a range of abnormalities in *Tmsb4x*^*−/y*^ and *Tmsb4x*^*+/y*^ mice injected with NTS including collapse of capillary loops, segmental or global glomerulosclerosis, adhesion of the glomerular tuft to the Bowman capsule, and glomerular epithelial hyperplasia lesions, a feature of early crescent formation in this model.^34^ Semiquantitative histologic scoring (Supplementary Figure S6A–E) by 2 blinded observers revealed that *Tmsb4x*^*+/y*^ mice injected with NTS had a significantly increased mean glomerular score compared with *Tmsb4x*^*+/y*^ mice without disease (*P* < 0.001) (Figure 3g–j). Glomeruli of *Tmsb4x*^*−/y*^ administered NTS had an even higher glomerular mean score that was significantly greater than that in *Tmsb4x*^*+/y*^ mice with NTS nephritis (*P* < 0.05). This was associated with an increased proportion of sclerotic glomeruli and an increased incidence of epithelial hyperplasia lesions in *Tmsb4x*^*−/y*^ compared with *Tmsb4x*^*+/y*^ mice administered NTS (Supplementary Figure S6F).

**Figure S7:**
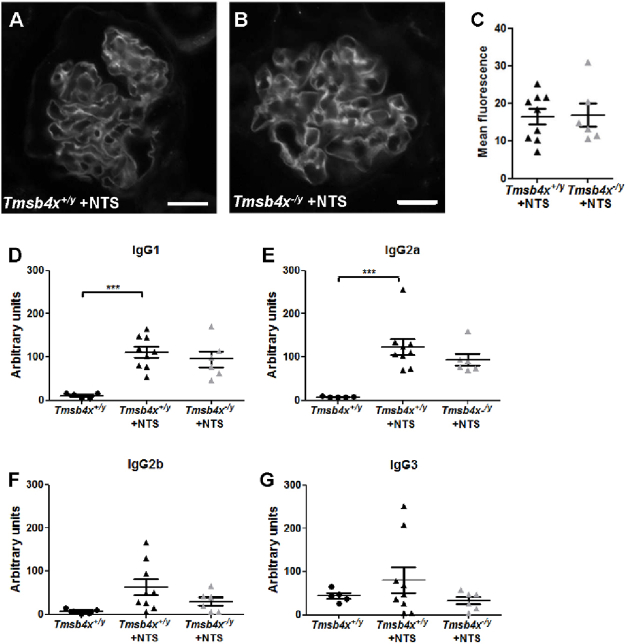
Representative images showing sheep IgG immunoreactivity in glomeruli of *Tmsb4x*^*+/y*^ (**A**) and *Tmsb4x*^*−/y*^ (**B**) mice injected with nephrotoxic serum (NTS). (**C**) Quantification of mean fluorescence in 30 glomeruli per sample (*Tmsb4*^*+/y*^ + NTS, *n* = 9; *Tmsb4x*^*−/y*^ + NTS, *n* = 6). Plasma titers of murine IgG subclasses (**D**, IgG1; **E**, IgG2a; **F**, IgG2b; **G**, IgG3) against sheep IgG in control, not immunized *Tmsb4x*^*+/y*^ mice (*n* = 5) and in *Tmsb4x*^*+/y*^ (*n* = 9) and *Tmsb4x*^*−/y*^ (*n* = 6) mice 21 days after NTS injection. Data are presented as mean ± SEM. Bar = 20 μm, *P ≤ 0.05, ** *P* ≤ 0.01, *** *P* ≤ 0.001.

We examined whether the difference in disease severity between *Tmsb4x*^*−/y*^ and *Tmsb4x*^*+/y*^ mice administered NTS could be due to a decrease in binding of the antiglomerular antibody but found no difference in the amount of sheep IgG deposited within the glomerulus (Supplementary Figure S7A–C). We assessed whether the lack of *Tmsb4x* changes the humoral immune response to sheep IgG. NTS injection led to significantly increased production of circulating murine IgG1 and IgG2a, but not IgG2b or IgG3, against sheep IgG compared with *Tmsb4x*^*+/y*^ mice without disease. However, there was no difference in the plasma titers of any of the IgG subclasses between *Tmsb4x*^*−/y*^ and *Tmsb4x*^*+/y*^ mice administered NTS (Supplementary Figure S7D–G).

### Changes in podocyte distribution in *Tmsb4x*^*−/y*^ glomeruli after NTS nephritis

**Figure S8:**
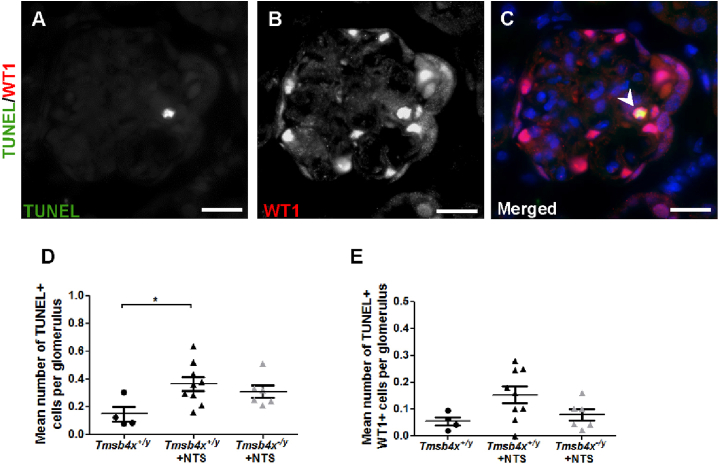
Representative image showing staining for TUNEL (**A**) and WT-1 (**B**) in the glomerulus. A TUNEL-positive podocyte is identified by an arrowhead (**C**). The number of TUNEL-positive cells (**D)** and TUNEL-positive WT-1–positive cells per glomerulus was counted in 50 glomeruli per sample, and the average number was calculated (*Tmsb4x*^*+/y*^, *n* = 4; *Tmsb4x*^*+/y*^, + NTS, *n* = 9; *Tmsb4x*^*−/y*^ + NTS, *n* = 6). Data are presented as mean ± SEM. Bar = 20 μm. *P ≤ 0.05.

**Figure 4:**
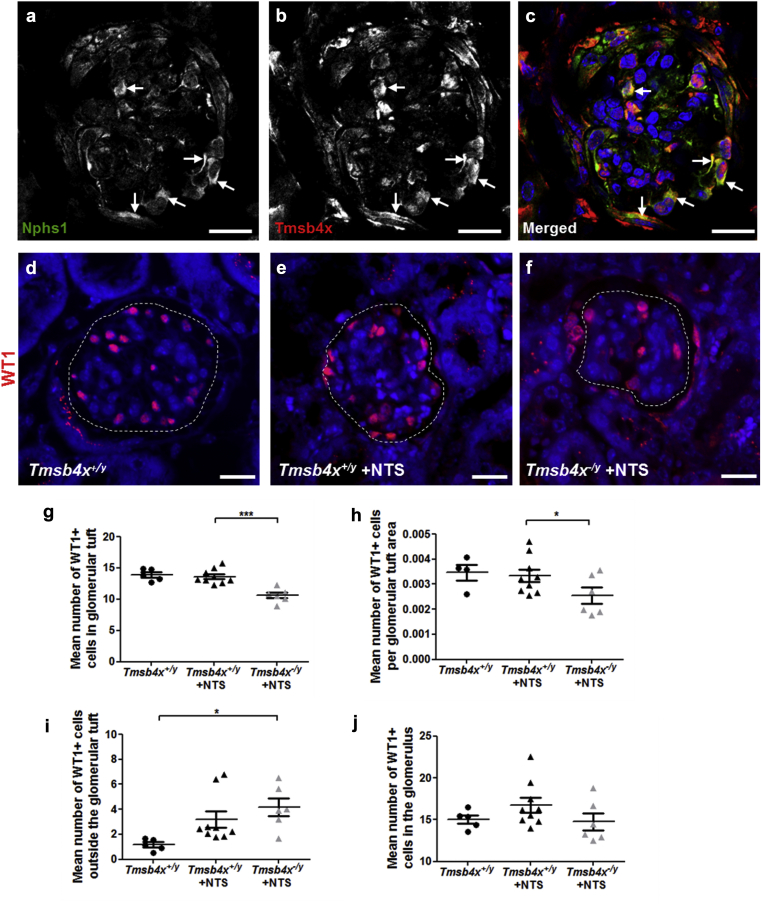
**Podocyte assessment in *Tmsb4x***^***+/y***^**and*Tmsb4x***^***−/y***^**mice after induction of nephrotoxic nephritis.** Representative image of nephrin (Nphs1; podocytes) (**a**) and Tmsb4x (**b**) staining in the adult wild-type mouse glomerulus 21 days after injection of nephrotoxic serum (NTS). Cells positive for both Nphs1 (green) and Tmsb4x (red) are indicated by arrows in the merged image (**c**). Representative images of glomeruli from *Tmsb4x*^*+/y*^ (**d**), *Tmsb4x*^*+/y*^ + NTS (**e**), and *Tmsb4x*^*−/y*^ + NTS (**f**) mice stained for WT1. The glomerular tuft areas are indicate the dotted lines. (**g–j**) Graphs showing the number of WT1+ cells in the glomerular tuft (**g**), the number of WT1^+^ cells in the glomerular tuft normalized to the glomerular area (**h**), the number of WT1+ cells in the area of the glomerulus surrounding the glomerular tuft (**i**), and the number of WT1+ cells in the whole glomerulus (**j**). Cells were counted as 50 glomeruli per sample, except in (**h**), where cells were counted and normalized to the glomerular area as 15 glomeruli per sample. Data are presented as mean ± SEM. *Tmsb4x*^*+/y*^ (*n* = 5), *Tmsb4*^*+/y*^ + NTS (*n* = 9), and *Tmsb4x*^*−/y*^ + NTS (*n* = 6) mice. Bar = 20 μm. Tmsb4x, thymosin β_4_.

After NTS nephritis, we found that Tmsb4x still colocalized with Nphs1 (Figure 4a–c) and subsequently examined the effect that a lack of Tmsb4x had on podocytes in this model. First, we quantified Wilms Tumor 1 postive (WT1^+^) podocyte numbers,^35^ inside and outside of the glomerular tuft (Figure 4d–f). The number of glomerular tuft WT1^+^ cells was unchanged in *Tmsb4x*^*+/y*^ mice after NTS injection compared with mice without disease. However, NTS administration to *Tmsb4x*^*−/y*^ mice significantly reduced the number of WT1^+^ glomerular tuft cells compared with *Tmsb4x*^*+/y*^ mice with NTS nephritis (*P* < 0.001) (Figure 4g). This finding persisted after normalizing the WT1^+^ cell number to the glomerular tuft area (*P* < 0.05) (Figure 4h). In contrast, there was an increased number of WT1^+^ cells outside the glomerular tuft in both *Tmsb4x*^*+/y*^ and *Tmsb4x*^*−/y*^ mice after NTS administration; this was more prominent and significantly different in the *Tmsb4x*^*−/y*^ animals (*P* < 0.05 compared with *Tmsb4x*^*+/y*^ without disease) (Figure 4i). The total number of podocytes in the whole glomerulus did not differ between any of the groups (Figure 4j), suggesting that lack of Tmsb4x leads to podocyte redistribution from the glomerular tuft toward the Bowman capsule rather than affecting podocyte cell death. To support this, we assessed podocyte apoptosis using terminal deoxynucleotidyltransferase–mediated dUTP nick end-labeling (TUNEL)^36^ in combination with WT1 staining (Supplementary Figure S8A–C). Administration of NTS increased the number of glomerular apoptotic cells compared with healthy mice, and this effect was significant in *Tmsb4*^*+/y*^ mice with glomerular disease (*P* < 0.05) (Supplementary Figure S8D). However, the number of TUNEL^+^/WT1^+^ cells was not significantly different between any of the groups (Supplementary Figure S8E).

### Lack of Tmsb4x induces migration and modulates the cytoskeleton of podocytes *in vitro*

**Figure 5:**
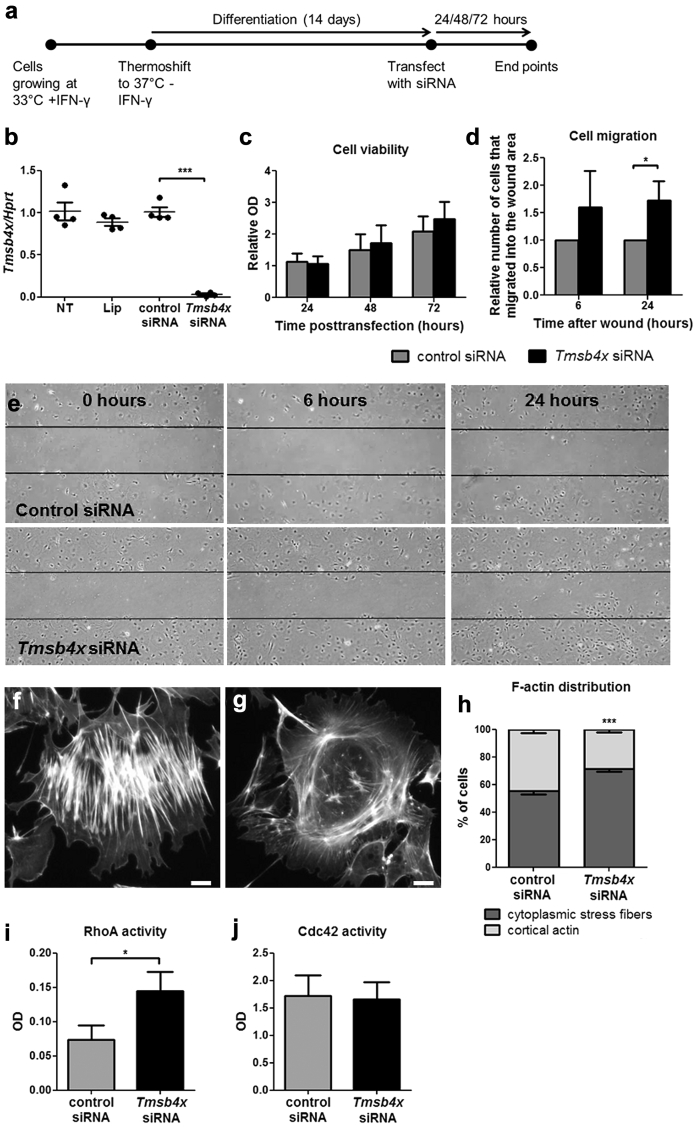
**Effects of downregulating endogenous *Tmsb4x* expression in podocytes *in vitro*.** (**a**) Podocytes grown *in vitro* under permissive conditions were differentiated for 14 days before transfecting them with control siRNA or siRNA targeting *Tmsb4x*. (**b**) Quantification of *Tmsb4x* mRNA levels in podocytes 48 hours after transfection. (**c**) Cell viability after knockdown of endogenous *Tmsb4x* was assessed by MTT assay. (**d**) Podocyte migration after knockdown of endogenous *Tmsb4x* was assessed by a wound-healing assay, and the number of cells that migrated into the wound area was counted. (**e**) Representative images of podocytes transfected with control or *Tmsb4x* siRNA 0, 6, and 24 hours after wound formation. Representative images showing a podocyte with cytoplasmic stress fiber F-actin distribution (**f**) or cortical F-actin distribution (**g**). The percentage of cells with predominantly cytoplasmic stress fibers or cortical actin formation was quantified 48 hours after transfection (**h**). Quantification of active RhoA (**i**) and active Cdc42 (**j**) 48 hours after transfection. All experiments were repeated 3 to 4 times, and the data are presented as mean ± SEM. *P ≤ 0.05, ***P ≤ 0.001. IFN-γ, interferon-γ; Lip, lipofectamine; OD, optical density; siRNA, small, interfering RNA; TB, Tmsb4x, thymosin β_4_.

We postulated that the redistribution of podocytes in nephrotoxic nephritis may be due to changes in cell migration driven by lack of Tmsb4x. Therefore, we transfected cultured differentiated mouse podocytes^37^ with siRNA against *Tmsb4x* (Figure 5a); this resulted in >90% knockdown in *Tmsb4x* levels (Figure 5b) (*P* < 0.001). Knockdown of endogenous *Tmsb4x* did not affect podocyte viability (Figure 5c) but increased the number of cells that migrated into the wound area in a wound-healing assay (*P* < 0.05) (Figure 5d and e). Because the cytoskeleton is essential for cell movement,^38^ we visualized podocyte actin by phalloidin staining and classified the filament organization as either cytoplasmic stress fibers (Figure 5f) or cortical actin (Figure 5g). Knockdown of endogenous *Tmsb4x* significantly increased the percentage of cells with stress actin fiber organization (*P* < 0.001) (Figure 5h). Finally, we assessed the effects of Tmsb4x knockdown on the activation of RhoA and Cdc42, which regulate actin dynamics and cell migration.^39^ There was increased RhoA activity in podocytes transfected with *Tmsb4x* siRNA compared with control siRNA (*P* < 0.05) (Figure 5i), whereas Cdc42 activity was unaffected (Figure 5j).

### Macrophage accumulation and increased fibrosis in *Tmsb4x*^*−/y*^ glomeruli after NTS nephritis

**Figure 6:**
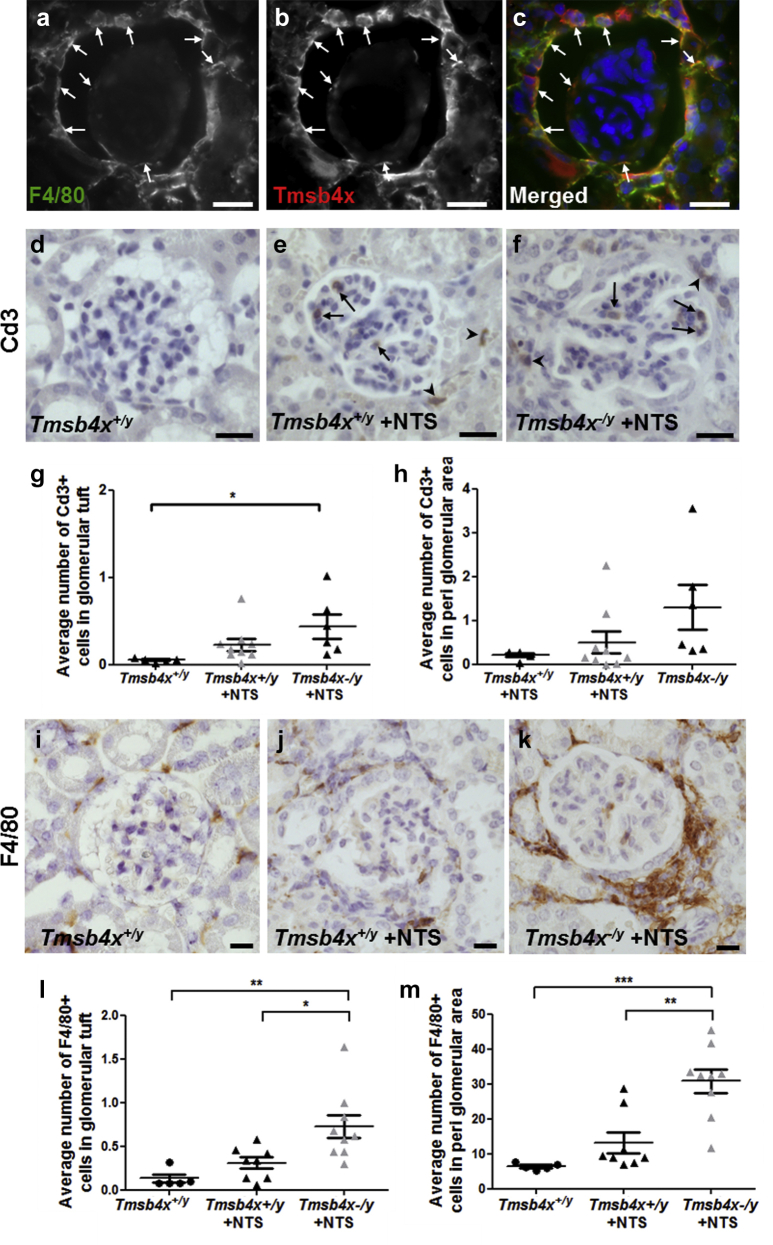
**Assessment of inflammation in nephrotoxic nephritis.** Representative images of F4/80 (macrophages) (**a**) and thymosin β_4_ (Tmsb4x) staining (**b**). Cells positive for both F4/80 (green) and Tmsb4x (red) are shown in the merged image (**c**) and are indicated by arrows. Images were taken by fluorescent microscopy. Bar = 20 μm. Representative images showing Cd3 (T-cell marker) staining in the glomerular tuft (arrows) and in the periglomerular area (arrowheads) of *Tmsb4x*^*+/y*^ controls (**d**) and *Tmsb4x*^*+/y*^ (**e**) and *Tmsb4x*^*−/y*^ (**f**) mice 21 days after administration of nephrotoxic serum (NTS). The number of Cd3+ cells in the glomerular tuft (**g**) and in the periglomerular area (**h**) was counted as 50 glomeruli per sample, and the average number was calculated (*Tmsb4x*^*+/y*^, *n* = 5; *Tmsb4x*^*+/y*^ + NTS, *n* = 9; *Tmsb4x*^*−/y*^ + NTS, *n* = 6). Representative images showing F4/80 (activated macrophage marker) staining in the glomerular tuft and the periglomerular area of *Tmsb4x*^*+/y*^ control (**i**), *Tmsb4x*^*+/y*^ + NTS (**j**), and *Tmsb4x*^*−/y*^ + NTS (**k**) mice. The number of F4/80+ cells in the glomerular tuft (**l**) and in the periglomerular area (**m**) was counted as 50 glomeruli per sample, and the average number was calculated (*Tmsb4x*^*+/y*^, *n* = 5; *Tmsb4x*^*+/y*^ + NTS, *n* = 8; *Tmsb4x*^*−/y*^ + NTS, *n* = 6). Data are presented as mean ± SEM. *P ≤ 0.05, **P ≤ 0.01, ***P ≤ 0.001. Bar = 20 μm. Tmsb4x, thymosin β_4_.

Tmsb4x is expressed in macrophages^18^, ^40^ and reduces inflammation in several disease settings.^10^, ^16^, ^18^, ^41^ As immune cell infiltration plays a critical role in the initiation and progression of crescentic glomerulonephritis,^42^, ^43^, ^44^ we decided to examine this in the NTS nephritis model. We found expression of Tmsb4x in F4/80^+^ macrophages surrounding the glomeruli and occasionally within the glomerular tuft (Figure 6a–c) and went on to examine the effect of *Tmsb4x* loss on glomerular inflammation in our experimental model.

**Figure S9:**
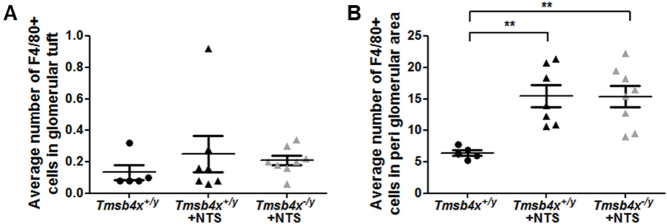
The number of F4/80+ cells in the glomerular tuft (**A**) and in the periglomerular area (**B**) 7 days after administration of nephrotoxic serum (NTS) was counted in 50 glomeruli per sample, and the average number was calculated (*Tmsb4x*^*+/y*^, *n* = 5; *Tmsb4x*^*+/y*^ + NTS+, *n* = 6; *Tmsb4x*^*−/y*^ + NTS, *n* = 8). Data are presented as mean ± SEM. **P ≤ 0.01.

**Figure S10:**
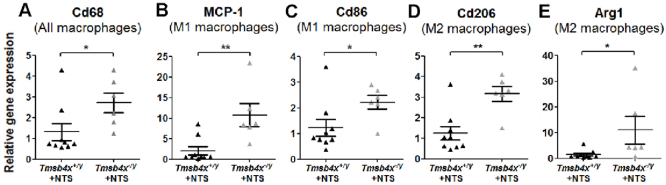
Quantification of *Cd68* (**A**), *Mcp1* (**B**), *Cd86* (**C**), *Cd206* (**D**), and *Arg1* (**E**) mRNA in whole-kidney homogenates of *Tmsb4*^*+/y*^ + NTS (*n* = 9) and *Tmsb4x*^*−/y*^ + NTS (*n* = 6) mice. NTS = nephrotoxic serum.

We measured the number of Cd3^+^ (T cells) and F4/80^+^ cells in *Tmsb4x*^*−/y*^ and *Tmsb4x*^*+/y*^ glomeruli. Twenty-one days after NTS injection, there was a significant increase in Cd3^+^ cells in the glomerular tuft of *Tmsb4x*^*−/y*^ mice compared with *Tmsb4x*^*+/y*^ mice without disease (*P* < 0.05) but no significant difference when comparing *Tmsb4x*^*−/y*^ and *Tmsb4x*^*+/y*^ mice with nephrotoxic nephritis (Figure 6d–g). There was also no difference in the number of periglomerular Cd3^+^ cells between experimental groups (Figure 6h). Seven days after NTS administration, the number of F4/80^+^ glomerular tuft cells was similar in all experimental groups, but were significantly increased in the periglomerular area of both NTS-injected *Tmsb4x*^*+/y*^ and *Tmsb4x*^*−/y*^ mice compared with healthy mice (*P* < 0.01) (Supplementary Figure S9A and B). The accumulation of F4/80^+^ cells persisted in *Tmsb4x*^*−/y*^ mice 21 days after NTS administration, with increased numbers in both the glomerular tuft and periglomerular area compared with *Tmsb4x*^*+/y*^ mice with or without disease (*P* < 0.01) (Figure 6i–m). mRNA levels of the pan-macrophage marker *Cd68* were also significantly higher 21 days after NTS injection in whole-kidney homogenates obtained from *Tmsb4x*^*−/y*^ compared with *Tmsb4x*^*+/y*^ mice (Supplementary Figure S10A). Cd68 is expressed by all macrophages, but these comprise a diverse group that includes a broad spectrum of cellular phenotypes, often characterized as pro-inflammatory (M1 type) and tissue repair (M2 type) macrophages. We quantified the mRNA levels of M1 (*Mcp1*, *Cd86*) and M2 markers (*Cd206*, *Arg1*) and found all of these genes were significantly upregulated in *Tmsb4x*^*−/y*^ mice compared with *Tmsb4x*^*+/y*^ mice after NTS administration (Supplementary Figure S10B–E). This suggests that there is a global increase in macrophages in *Tmsb4x*^*−/y*^ kidneys after NTS rather than a shift toward an M1 or M2 phenotype.

**Figure 7:**
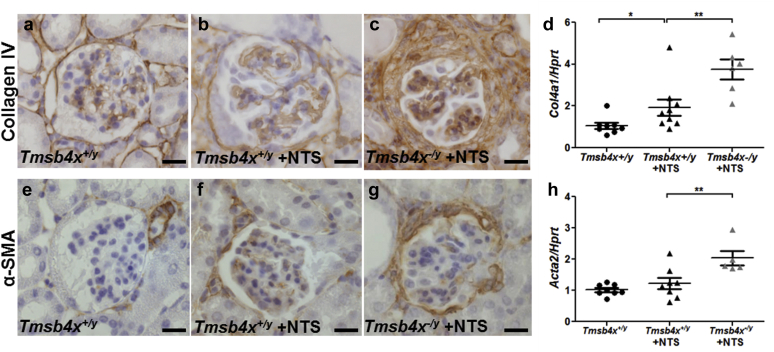
**Assessment of fibrosis in nephrotoxic nephritis.** Representative images showing collagen IV staining in glomeruli of *Tmsb4x*^*+/y*^ (**a**), *Tmsb4x*^*+/y*^ + nephrotoxic serum (NTS) (**b**), and *Tmsb4x*^*−/y*^ + NTS (**c)** mice. (**d**) Quantification of collagen IV (*Col4a1*) mRNA levels in whole-kidney homogenates of *Tmsb4x*^*+/y*^ (*n* = 8), *Tmsb4x*^*+/y*^ + NTS (*n* = 9), and *Tmsb4x*^*−/y*^ + NTS (*n* = 6) mice. Representative images showing α-smooth muscle actin (α-SMA) staining in glomeruli of *Tmsb4x*^*+/y*^ (**e**) *Tmsb4x*^*+/y*^ + NTS (**f**) and *Tmsb4x*^*−/y*^ + NTS (**g**) mice. (**h**) Quantification of α-SMA (*Acta2*) mRNA levels in whole-kidney homogenates of *Tmsb4x*^*+/y*^ (*n* = 8), *Tmsb4x*^*+/y*^ + NTS (*n* = 8), and *Tmsb4x*^*−/y*^ + NTS (*n* = 5) mice. Data are presented as mean ± SEM. *P ≤ 0.05, **P ≤ 0.01. Bar = 20 μm. Tmsb4x, thymosin β_4_.

Finally, the increased accumulation of macrophages in the periglomerular area in *Tmsb4x*^*−/y*^ mice with NTS was associated with increased periglomerular fibrosis, as shown by increased staining for both collagen IV (Figure 7a–c) and α-smooth muscle actin (Figure 7e–g) in sections from *Tmsb4x*^*−/y*^ compared with *Tmsb4x*^*+/y*^ mice injected with NTS along with increased whole-kidney mRNA levels of *Col4a1* (*P* < 0.05) (Figure 7d) and *Acta2* (*P* < 0.05) (Figure 7h).

## Discussion

In this study, we found endogenous Tmsb4x was not required to maintain glomerular structure and function in healthy adult mice. However, in an experimental model of NTS nephritis, glomerular disease was exacerbated in mice lacking *Tmsb4x* accompanied by changes in the distribution of podocytes within the glomerulus, increased periglomerular macrophage accumulation, and enhanced fibrosis. These findings provide the first evidence that endogenous Tmsb4x modifies glomerular injury, likely having a protective role acting as a brake to slow disease progression.

We showed that Tmsb4x is expressed in developing and adult mouse glomeruli, predominantly localized to podocytes. Previous studies also found Tmsb4x in glomerular podocytes,^25^ but others reported the complete absence of Tmsb4x in the glomeruli of human fetal and adult kidneys^45^ and rat kidneys.^26^ These discrepancies may be due to differences in the antibodies and fixation methods used.^46^ Importantly, we obtained similar results for both Tmsb4x mRNA and protein and confirmed the specificity of antibody staining using tissue from *Tmsb4x*^*−/y*^ mice as an additional negative control.

Given that Tmsb4x plays a role in actin binding,^6^ we initially hypothesized that the lack of endogenous Tmsb4x might disrupt the highly branched architecture of glomerular podocytes and impair renal function.^3^ However, the lack of *Tmsb4x* did not affect glomerular morphology or podocyte architecture of normal healthy mice *in vivo*. We found upregulation of *Cfn1*, which severs actin filaments, in whole *Tmsb4x*^*−/y*^ kidneys. This could partly compensate for the lack of Tmsb4x and maintain actin dynamics^47^; however, *Cfn1* was not specifically altered in podocytes lacking Tmsb4x, thus making this unlikely.

A significant finding of our study was that severity of glomerular disease induced by NTS was greater in *Tmsb4x*^*−/y*^ mice compared with wild-type littermates. We postulate that endogenous Tmsb4x has a protective role in the setting of NTS, a prediction supported by a study showing that exogenous administration of Ac-SDKP ameliorated rat glomerulonephritis.^22^ However, we found that whole-mouse kidney *Tmsb4x* mRNA levels were unchanged with NTS nephritis, and this was mirrored when we assessed *TMSB4X* mRNA levels in glomerular and tubulointerstitial extracts from human kidneys affected by rapidly progressive glomerulonephritis or lupus nephritis. In contrast, a previous study in rat remnant kidneys in which nephron loss results in focal segmental glomerulosclerosis showed that Tmsb4x protein levels were significantly increased in sclerotic versus normal glomeruli.^26^ The discrepancy between these findings may be due to the different renal injury models and time points examined.

There are likely to be multiple mechanisms by which the lack of endogenous *Tmsb4x* results in increased glomerular injury in our experimental model. During nephrotoxic nephritis, podocytes switch from a terminally differentiated cell to a migratory cell that forms bridges between the glomerular tuft and the Bowman capsule^34^ and populates glomerular crescents.^30^, ^48^ The lack of *Tmsb4x* increased the number of glomeruli with adhesion of the glomerular tuft to the Bowman capsule and glomerular epithelial hyperplasia lesions, a feature of early crescent formation in this model.^34^ We also found that there was a redistribution of podocytes from the glomerular tuft, where they contribute to filtration barrier integrity toward the Bowman capsule. Our *in vitro* data demonstrate that downregulation of endogenous *Tmsb4x* in podocytes increases migration, and we predict that this may promote their redistribution in the nephrotoxic nephritis model. The increased podocyte migration was associated with increased actin stress fibers and activation of RhoA, which has been linked to podocyte stress fiber formation.^49^, ^50^ Moreover, podocyte-specific overexpression of RhoA induces proteinuria,^50^, ^51^ whereas RhoA inhibition improves renal injury in mouse models of nephrectomy^52^ and nephrotoxic nephritis,^53^ demonstrating the functional importance of this pathway in glomerular function. Other studies have shown that RhoA activation inhibits podocyte migration,^54^ but these experiments used a constitutively active form of RhoA permanently in the guanosine triphosphate-bound state. This would result in a high degree of RhoA activity, which has been associated with inhibition of migration.^55^ In contrast, *Tmsb4x* knockdown led to a 2-fold upregulation of RhoA activity in podocytes. It has been postulated that this lesser degree of RhoA activation promotes contractile stress fiber formation, facilitating cell detachment in migrating cells^39^ and enhancing lamellipodia formation driving cell motility.^55^ It was previously reported that activation of other Rho GTPases, Cdc42 and Rac1, may increase podocyte migration.^56^ However, we found that downregulation of endogenous *Tmsb4x* did not affect Cdc42 activation in podocytes. Rac1 activity was not assessed in this study, and it would be interesting to explore its involvement in the future.

Tmsb4x is also expressed in macrophages,^18^, ^40^ including in our nephrotoxic nephritis model, but its precise function has yet to be determined. It could be postulated that loss of the macrophage *Tmsb4x* may regulate the actin cytoskeleton, which has been implicated in both phenotypic polarization^57^ and migration.^58^ In our study, the loss of *Tmsb4x* did not alter macrophage polarization or the number of activated macrophages found in the glomerular area in the early stages of nephrotoxic nephritis. However, the number of activated macrophages in the periglomerular area at the late stage of the disease was increased in *Tmsb4x*^*−/y*^ mice, suggesting a deficiency in the resolution of inflammation resulting in persistent macrophage accumulation. Macrophage accumulation may result from an absence of the Tmsb4x-derivative thymosin β_4_ sulfoxide, which has been shown to disperse inflammatory macrophages at the injury site in zebrafish and mouse models of heart injury.^59^ We also found that periglomerular fibrosis was enhanced in *Tmsb4x*^*−/y*^ mice after NTS administration compared with wild-type mice. This may represent a secondary effect of enhanced inflammation and glomerular damage. However, previous studies showed that Tmsb4x can alter both plasminogen activator inhibitor-1 and transforming growth factor-β_1_,^18^, ^27^ both of which are drivers of fibrosis and play important roles in the progression of nephrotoxic nephritis.^60^, ^61^

In summary, we have provided the first evidence that the lack of endogenous *Tmsb4x* does not affect healthy glomeruli but exacerbates renal function impairment, periglomerular inflammation, and fibrosis in the context of nephrotoxic nephritis. These findings suggest that modulating Tmsb4x could be a potential therapeutic target in immune-mediated glomerular disease.

## Methods

### Experimental animals and procedures

C57Bl/6 hemizygous null male mice (*Tmsb4x*^*−/y*^) were bred with heterozygous *Tmsb4x*^*+/−*^ adult female mice to generate male wild-type (*Tmsb4x*^*+/y*^) and null mice.^27^ To induce glomerular disease, *Tmsb4*^*+/y*^ and *Tmsb4x*^*−/y*^ mice were preimmunized by subcutaneous injection of sheep IgG (250 μg) in complete Freund’s adjuvant, followed by i.v. administration of sheep NTS (250 μl) 5 days later.^62^ All procedures were approved by the UK Home Office.

### Renal function

Urine was collected from mice by housing them individually in metabolic cages. Blood samples were collected from the lateral saphenous vein. Albumin concentrations were measured by enzyme-linked immunosorbent assay^28^, ^63^ (Bethyl Laboratories, Montgomery, TX). Urinary and plasma creatinine concentrations were measured using isotope dilution electrospray mass spectrometry.^64^ Creatinine clearance (μl/min per gram of body weight) was derived from the formula urinary creatinine × urine volume × 1440 min^−1^ × plasma creatinine^−1^ × body weight (g)^−1^.^63^ Blood urea nitrogen was assessed using a commercially available assay kit, validated in mice (BioAssay Systems, Hayward, CA).^65^

### Histologic analysis and immunohistochemistry

Kidneys were fixed in 4% paraformaldehyde and embedded in paraffin, and 5-μm sections were cut and stained with periodic acid–Schiff reagent. Fifty glomeruli per sample were scored by 2 blinded observers using the following system: 0, normal glomerular structure; 1, increased mesangial matrix deposition and hypercellularity with some loss of capillary loops; 2, increased matrix deposition and focal areas of sclerosis; 3, >50% of glomerulus sclerotic with very few capillary loops; 4, >75% of glomerulus sclerotic and the presence of glomerular epithelial hyperplasia lesions (Supplementary Figure S5). An average score was obtained for each kidney.

Immunohistochemistry or immunofluorescence was performed^66^ using antibodies against Tmsb4x (A9520, Immundiagnostik, Bensheim, Germany), collagen IV (ab19808, Abcam, Cambridge, UK), Cd3 (ab16669, Abcam), α-smooth muscle actin (M0851, Dako, Ely, UK), F4/80 (MCA497R, AbD Serotec, Oxford, UK), Nphs1 (GP-N2, Progen, Heidelberg, Germany), nestin (NB100-1604, Novus Biologicals, Littleton, CO), WT1 (AP15857PU-S, Acris Antibodies, Herford, Germany), Cd31 (MA3105, Thermo Fisher Scientific, Waltham, MA), and sheep IgG (A11016, Thermo Fisher Scientific). CD3^+^ and F4/80^+^ cells were counted in 50 glomeruli per sample. To assess glomerular sheep IgG deposition, mean fluorescence intensity was measured using ImageJ^67^ (30 glomeruli/sample). The number of WT1^+^ cells found within or outside (in glomerular crescents or lining the Bowman capsule) the glomerular tuft was counted in 50 consecutive glomeruli per sample. To account for any changes in the glomerular tuft area, the number of WT1^+^ cells in the glomerular tuft was normalized to the glomerular area (measured using ImageJ^67^) in 15 glomeruli per sample. Apoptosis was identified using TUNEL (Roche, Burgess Hill, UK). The number of TUNEL^+^ and WT1^+^/TUNEL^+^ cells was counted in 50 glomeruli per sample.

### Measurement of murine IgG subclasses specific for sheep IgG

The titers of murine IgG subclasses specific for sheep IgG were measured by enzyme-linked immunosorbent assay in plasma, as described,^68^ using alkaline phosphatase subclass-specific antibodies for IgG1, IgG2b, and IgG3 (SouthernBiotech, Birmingham, AL) and IgG2a (Bethyl Laboratories).

### *In situ* hybridization

*In situ* hybridization on paraffin sections was performed as described,^27^ using a digoxigenin-labeled antisense riboprobe specific for the 3′UTR of *Tmsb4x*, alongside a sense control.

### Electron microscopy

For transmission electron microscopy, kidney cortex specimens (1 mm^3^) were postfixed in osmium tetroxide, dehydrated in acetone, and embedded in epoxy resin. Ultrathin sections were stained with uranyl acetate and lead citrate and examined.

### Quantitative real-time polymerase chain reaction

RNA was extracted from mouse whole-kidney or glomerular extracts (isolated by Dynabeads [Thermo Fisher Scientific]^28^); 500 ng was used to prepare cDNA (iScript kit, Bio-Rad, Hemel Hempstead, UK), and quantitative real-time polymerase chain reaction was performed as described^28^ with *Hprt* as a housekeeping gene. All measurements were performed in duplicate. Renal biopsy specimens from patients with rapidly progressive glomerulonephritis (*n* = 12), lupus nephritis (*n* = 12), and living donor controls (*n* = 7) were collected within the framework of the European Renal cDNA Bank–Kroener–Fresenius Biopsy Bank^69^ after informed consent and local ethics committee approval. Unfixed tissue was transferred to RNase inhibitor and manually microdissected into glomerular and tubulointerstitial compartments. Total RNA was isolated and quantitative real-time polymerase chain reaction performed as reported,^69^ with 18S rRNA (Applied Biosystems, Foster City, CA) as the reference gene. Primer details are available on request.

### Cell culture

Mouse podocytes^37^ were cultured as described^70^ and allowed to differentiate for 14 days. Cells were transfected with 10 nM siRNA specific for *Tmsb4x* or with a nontargeting control (both from Santa Cruz Biotechnology, Dallas, TX) using the transfection reagent Lipofectamine RNAiMAX (Thermo Fisher Scientific) according to the manufacturer’s instructions.

Cell viability was assessed 24, 48, and 72 hours post-transfection using the methyltetrazolium assay. To assess migration, podocytes were plated to confluence, and a scratch was created using a pipette tip. Images (4 fields per condition) were taken 0, 6, and 24 hours later and the number of cells that migrated into the wound area counted. To visualize F-actin filaments 48 hours post-transfection, podocytes were fixed in 4% paraformaldehyde and 4% sucrose and stained with AlexaFluor-594 phalloidin (Thermo Fisher Scientific). The arrangement of actin filaments (either cytoplasmic stress fibers or cortical actin) was assessed in 30 cells per condition. RhoA and Cdc42 activity was quantified in podocyte lysates by G-LISA Small G-protein Activation Assays (Cytoskeleton, Denver, CO).

Glomeruli from *Tmsb4x*^*+/y*^ and *Tmsb4x*^*−/y*^ mice were isolated by Dynabeads (Thermo Fisher Scientific)^28^ and cultured in Matrigel-coated plates (Corning, Tewksbury, MA) in Dulbecco’s modified Eagle’s medium:F12 with 10% fetal calf serum, 1% Insulin-Transferrin-Selenium, and 100 μg/ml penicillin (Thermo Fisher Scientific). On day 7, when podocytes had grown out of the glomeruli, they were detached using trypsin–ethylenediamine tetraacetic acid and separated from glomeruli using 40-μm cell strainers (Corning). Primary podocytes obtained with this method were >90% pure, as judged by cell morphology and staining using podocyte (nephrin, nestin) markers.

### Statistical methods

All samples were assessed blinded to treatment. Data are presented as mean ± SEM and were analyzed using GraphPad Prism (GraphPad Software, La Jolla, CA). When differences between 2 groups were evaluated, data were analyzed using a *t* test. When ≥3 groups were assessed, 1-way analysis of variance with Bonferroni’s multiple comparison *post hoc* tests were used. Data affected by 2 variables were analyzed using 2-way analysis of variance with Bonferroni’s multiple comparison *post hoc* tests. Statistical significance was accepted at *P* ≤ 0.05.

## Disclosure

All the authors declared no competing interests.

## Supplementary Material

Table S1 Proportions of *Tmsb4x*^*+/−*^, *Tmsb4x*^*−*/*−*^, *Tmsb4x*^*+/y*^, and *Tmsb4x*^*−/y*^ mice born after crossing adult male *Tmsb4x*^*−/y*^ mice with *Tmsb4x*^*+/−*^ adult female mice.
