# Supplementary material for: Loss of endogenous thymosin β4 accelerates glomerular disease
Source: Kidney Int. 2016 Nov;90(5):1056–70. doi: 10.1016/j.kint.2016.06.032 (PMC5073078; doi:10.1016/j.kint.2016.06.032)
Supplement: Table S1 — Proportions of Tmsb4x+/−, Tmsb4x−/−, Tmsb4x+/y, and Tmsb4x−/y mice born after crossing adult male Tmsb4x−/y mice with Tmsb4x+/− adult female mice. [file mmc1.docx]

**Supplementary Table 1** Proportions of *Tmsb4x^+/-^, Tmsb4x*^-/-^, *Tmsb4x^+/y^* and *Tmsb4x^-/y^* mice born after crossing adult male *Tmsb4x^-/y^* mice with *Tmsb4x^+/-^* adult females.

| Genotype | *Tmsb4x^+/-^* | *Tmsb4x^-/-^* | *Tmsb4x^+/y^* | *Tmsb4x^-/y^* | Total | *Χ^2^* | *P* value |
| --- | --- | --- | --- | --- | --- | --- | --- |
| Expected | 25% | 25% | 25% | 25% |  |  |  |
| Observed | 92 (28%) | 74 (23%) | 85 (26%) | 76 (23%) | 327 | 2.554 | 0.4657 |
